# Single- and double-walled boron nitride nanotubes: Controlled synthesis and application for water purification

**DOI:** 10.1038/s41598-020-64096-z

**Published:** 2020-05-04

**Authors:** Hyunjin Cho, Jun Hee Kim, Jae Hun Hwang, Cheol Sang Kim, Se Gyu Jang, Cheol Park, Hunsu Lee, Myung Jong Kim

**Affiliations:** 1 Functional Composite Materials Research Center, Korea Institute of Science and Technology, 92, Chudong-ro, Bongdong-eup, Wanju, Jeollabuk-do 55324 Republic of Korea; 2 Department of Bionanosystem Engineering, Jeonbuk National University, 567, Baekje-daero, Deokjin-gu, Jeonju, Jeollabuk-do 54896 Republic of Korea; 3 Division of Mechanical Design Engineering, Jeonbuk National University, 567, Baekje-daero, Deokjin-gu, Jeonju, Jeollabuk-do 54896 Republic of Korea; 4 Advanced Materials and Processing Branch, NASA Langley Research Center, Hampton, Virginia 23681 USA; 5 Mutifunctional Structural Composite Research Center, Korea Institute of Science and Technology, 92 Chudong-ro, Bongdong-eup, Wanju, Jeollabuk-do 55324 Republic of Korea; 6 Security and Disruptive Technologies Research Centre, National Research Council Canada, 1200 Montreal Road, Ottawa Ontario, K1A 0R6 Canada; 7 Department of Chemistry, Gachon University, 1342 Seongnam-daero, Sujeong-gu, Seongnam-si, Gyeonggi-do 13120 Republic of Korea

**Keywords:** Synthesis and processing, Nanoscale materials

## Abstract

Research interest in boron nitride nanotubes (BNNTs) has increased after the recent success of large-scale BNNT syntheses using high-temperature-pressure laser ablation or high-temperature plasma methods. Nonetheless, there are limits to the application and commercialization of these materials because of the difficulties associated with their fine structural control. Herein, the growth kinetics of BNNTs were systemically studied for this purpose. The growth pressure of the nitrogen feed gas was varied while the growth temperature remained constant, which was confirmed by black body radiation measurements and calculations based on a heat loss model. Changing from the diffusion-limited regime to the supply-limited regime of growth kinetics based on the optimized BNNT synthesis condition afforded the control of the number of BNNT walls. The total amount of BNNTs possessing single and double walls was over 70%, and the BNNT surface area increased to 278.2 m^2^/g corresponding to small wall numbers and diameters. Taking advantage of the large surface area and high-temperature durability of the material, BNNTs utilized as a recyclable adsorbent for water purification. The efficiency of the BNNTs for capturing methylene blue particles in water was approximately 94%, even after three repetition cycles, showing the potential of the material for application in the filter industry.

## Introduction

Newly discovered materials such as fullerene, carbon nanotubes (CNTs), graphene, transition metal dichalcogenides, hexagonal boron nitride (h-BN), and boron nitride nanotubes (BNNTs) have provided great opportunities for scientific and technical improvements in various application industries in the past several decades^1–6^. Among these materials, one-dimensional (1D) BNNTs and two-dimensional (2D) h-BN are highly promising nanomaterials that have attracted the worldwide attention of many researchers and companies in various fields owing to their excellent physical and chemical properties and lightweight^6–12^. BNNTs and h-BN consist of boron and nitrogen covalently bonded in a honeycomb lattice and are structurally very similar to CNTs and graphene^10–15^. While carbon-based materials have high electrical conductivities due to the overlap of π orbitals^16,17^, BNNTs and h-BN composed of boron and nitrogen atoms are nearly insulators with ~5.8 eV bandgaps due to electron separation arising from the high electronegativity of the nitrogen atom^11,13,15,19,20,18,10^. In particular, BNNT has not only the unique 1D structure stable up to a temperature of 850 °C but also their superior mechanical properties and chemical resistance^7,9,10,19,21–23^. Therefore, BNNTs among BN materials have been applied to various applications, such as BNNT-polymer composites, BNNT-metal composites, and biological applications^7,9,10,12,15,21,24–33^. Additionally, since BNNTs have abundant B^10^ isotope, which has the largest scattering cross-section for neutron absorption among low Z atoms, they can be employed as a neutron shielding agent^24,34,35^.

Some research groups have attempted to increase BNNT production yield as well as to control the number of BNNT walls via various synthesis techniques^6,19,20,34,36–41^. However, arc-discharge and chemical vapor deposition (CVD) techniques have shown limited production yields despite synthesizing single-walled BNNTs (SWBNNTs) and double-walled BNNTs (DWBNNTs)^6,19,20,36,41^. Synthesis techniques based on the ball-milling methods have been introduced by several researchers as an alternative for the mass-production of BNNTs^10,20,39,40,42^. However, the resulting BNNTs often have bamboo-like structures with large diameters or are thick multi-walled BNNTs (MWBNNTs)^10,20,40^.

Recently, synthesis techniques that meet both the production yield and BNNT quality goals were discovered. The pressurized vapor condensation method using laser ablation (i.e., high temperature pressure (HTP) laser ablation) was first reported in 2009^34^, and the hydrogen-assisted boron nitride nanotube synthesis method using an induction plasma system followed in 2014^37^. In the same year, a synthesis technique by an extended-pressure inductively-coupled plasma system was also reported^38^. However, although these techniques not only have achieved both high production yields and high quality but also have shown SWBNNTs and DWBNNTs in the as-produced BNNTs, the fine structural control of BNNTs based on the growth mechanism remains in progress.

From a structural point of view, BNNTs can be classified into SWBNNTs, DWBNNTs, and MWBNNTs, which have both similarities and differences in their various properties. In particular, MWBNNTs can be considered as a series of SWBNNTs nested within one another. They may consist of at least three to approximately 100 concentric walls, and their diameters are approximately as large as 50 nm, while the diameters of SWBNNTs and DWBNNTs may be less than 6 nm. In the case of CNTs, single-walled CNTs (SWCNTs) have superior mechanical properties^43^ and larger surface areas per unit mass than other CNTs^44^. For these reasons, SWCNTs are preferred for various applications, and the developed synthesis techniques for high SWCNT yields have expanded their applicability to various research and industrial fields beyond composite materials. Thus, researchers in the BNNT field are interested in the selective synthesis of SWBNNTs and DWBNNTs because they have superior properties to those of MWBNNTs due to structural perfection and quantum confinement effects. However, studies on controlling the number of BNNT layers and reducing various impurities such as amorphous boron and h-BN remain in the early stages^20,34,37,40,45^.

Here, we investigated the growth kinetics of BNNTs in HTP laser ablation. The growth pressure of the nitrogen feed gas was varied while the growth temperature remained constant confirmed by black body radiation measurements and calculations based on a heat loss model including thermal conduction, convection, and radiation. The growth temperature was not affected by the nitrogen pressure because thermal radiation, which is insensitive to gas pressure, is the dominant heat transfer mechanism. By changing from the diffusion-limited regime to the supply-limited regime of growth kinetics, we observed a decrease in the number of BNNT walls. At a nitrogen pressure of 6 bar, the total amount of BNNTs possessing single and double walls was over 70%, and the surface area of the BNNTs increased to 278.2 m^2^/g corresponding to small wall numbers and diameters. Taking advantage of the large surface area and high-temperature durability of the SWBNNTs and DWBNNTs, BNNTs tested as a recyclable adsorbent for water purification. The efficiency of BNNTs for capturing methylene blue (MB) molecules in water was approximately 94%, even after three cycles of repetition.

## Experimental Section

### BNNT synthesis

BNNTs were synthesized using a continuous CO_2_ laser ablation system (laser oscillator, Laser C3000C, FANUC) and a unique chamber that was specially customized and manufactured^46^. The detailed operation technique was HTP laser ablation similar to that used in previously reported studies^34,47^. Boron filaments (Specialty Materials, Inc.) were used as a source of boron in BNNT synthesis, and high-pressure nitrogen gas from 6 bar to 14 bar was applied as a nitrogen source. First, when the continuous CO_2_ laser (laser power 1000 W, wavelength 10.6 μm) focuses on the tip of the boron filaments, they melt and form a molten boron ball. Nitrogen gas then dissociates on the surface of the molten boron ball and transforms into nitrogen atoms. Subsequently, the boron and nitrogen atoms rapidly react, leading to BNNT formation. The grown BNNTs were finally collected by a metal mesh positioned above the molten boron ball.

### Measurement of the absolute temperature of molten boron ball

According to Wien’s approximation, which is derived from Planck’s law of black body radiation, the surface temperature of the molten boron ball at the tip of the boron fiber can be measured from the radiated wavelength with the maximum intensity. During laser growth, the optical radiation from the ball was transferred to a spectrometer by optical fiber with a 600 μm core diameter via a lens system set on the surface of the window outside the chamber. The optical radiation was then measured with a camera (Istar DH334T-18H-13, Andor) integrated with a spectrometer (SR-500i, Andor). The wavelength range of the measurement was from 225 nm to 875 nm, which was set to cover the whole range of black body radiation based on the pre-measured radiation of the molten boron ball. The camera slit was set to 10 μm, the grating inside the spectrometer was set to 150 l/mm with a blaze at 500 nm, and the camera shutter was open for 10 ms for each measurement. For each experimental condition, ten shots of the signal were averaged to increase the signal-to-noise ratio.

### Purification of BNNTs (removal of amorphous boron elements)

The purification of BNNTs is an additional process for selectively collecting high-quality BNNTs and was performed to remove impurities generated during BNNT growth. As shown in Figure S5, the as-grown BNNTs in a quartz container were first placed in a box-type furnace and reacted at approximately 650 °C for 6 h in an air atmosphere. Since B_2_O_3_ and the pure BNNTs were strongly entangled with each other, tip probe sonication was utilized to efficiently separate them in methanol for 1 h, which selectively dissolved the B_2_O_3_. Subsequently, the pure, high-quality BNNTs were collected using a filtration method with a membrane filter (200 nm pore size). The prepared BNNT buckypaper was simply peeled off the membrane filter after drying for 24 h.

### Measurement of MB adsorption by BNNTs

The purified BNNTs (10 mg) were added to 30 mL of MB solution (concentration of 30 ppm), followed by stirring at 150 rpm. To evaluate the concentration of MB in the solution, UV-Vis absorption spectra were taken at 664 nm with increasing time from 10 min to 180 min. The adsorption rate, E, was calculated according to the equation $[E=(\frac{{C}_{0}-C}{{C}_{0}})\times 100 \% ]$ (C_0_: initial concentration, C: residual concentration). The performance was measured for three cycles to confirm the reusability of BNNTs, individually. After each cycle, samples were annealed at 400 °C for 1 h and used for further tests with the same process.

### Characterizations

The observation of BNNT growth was carried out by a high-speed camera (Phantom Miro M110, Vision Research) and an optical emission spectrometer (OES, Shamrock 500i, Andor). FE-SEM (Nova NanoSEM 450) and HR-TEM (Tecnai G2 F20, 200 kV) were utilized to observe the surface morphologies and layer structures of the BNNTs, respectively. The TEM samples for HRTEM and EELS analyses were prepared on a lacey carbon TEM grid (Lacey Carbon, 200 mesh, TH, Copper). Analyses of the crystallinity and elemental compositions of the BNNTs were carried out by Raman spectroscopy (Horiba, LabRAM HR UV-Visible-NIR, 514 nm), XPS (Thermo Scientific, K-Alpha), and EELS (Tecnai G2 F20, 200 kV). A UV-vis spectrophotometer (HP8453UV–vis spectroscopy system, Germany) was utilized to determine the concentration of MB.

## Results and discussion

**Figure 1:**
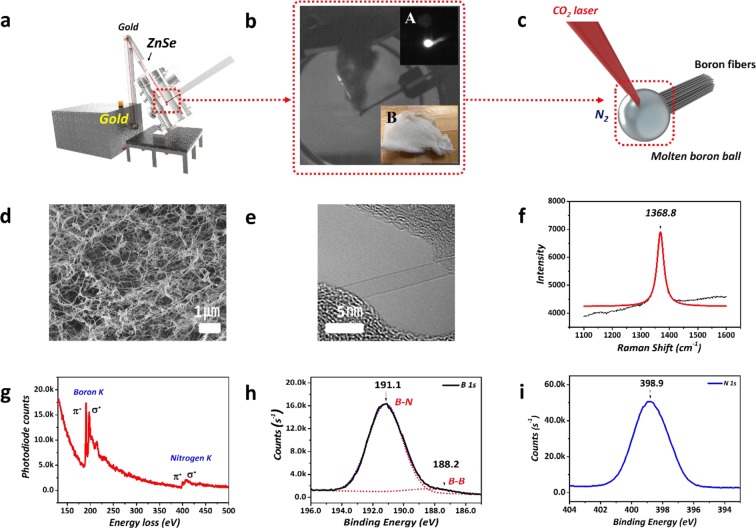
Growth and analysis results of BNNTs synthesized by laser ablation (**a**) Schematic of the customized laser ablation system and laser incident on the molten boron ball of the boron fiber. (**b**) Images of the synthesized BNNTs in the chamber; A is an image from the high-speed camera, and B is the BNNTs synthesized at 14 bar. (**c**) Schematic of BNNT growth. (**d**) SEM image of BNNTs. (**e**) TEM image of SWBNNTs. (**f**) Raman spectrum of BNNTs. (**g**) EELS spectrum of BNNTs. (**h,i**) XPS spectra of BNNTs.

Figure 1 is a scheme of the laser ablation system used for BNNT synthesis and various analysis results of the synthesized BNNTs. As shown in Figure 1a,c, a continuous 10.2 µm wavelength CO_2_ laser was applied for the BNNT synthesis, and the laser was effectively guided and concentrated into the end-edge of amorphous boron fibers in the synthesis chamber through gold mirrors and zinc selenide windows. Subsequently, the focused laser continuously melted the surface of the amorphous boron as a boron source, and 14 bar of high-pressure nitrogen was utilized as a nitrogen source. During growth, it is critical to form a molten boron ball with a constant size, as shown in inset A in Figure 1b, because the reaction area and temperature are crucial factors for BNNT growth. Inset B in Figure 1b is a photograph of the raw BNNTs synthesized at a nitrogen pressure of 14 bar, showing that the shape of the BNNTs was similar to that of white cotton fibers. The photograph in Figure 1b is a snapshot of the BNNTs in the middle of growth. The BNNTs were in the form of flexible fibers because they entangle and clump together during growth. The white color of the BNNTs indicated that optimized growth conditions were maintained. Ultimately, we synthesized a large amount of BNNTs (approximately 100 mg after 5 h). Additional Movie data (refer to Supplementary Information, Movie S1) shows the BNNT growth in detail recorded through a high-speed camera.

Figure 1d–i show the various analysis results of the BNNTs synthesized under optimal synthesis conditions. Figure 1d is a field emission scanning electron microscope (FE-SEM) image of the raw BNNTs, which confirmed that the entangled BNNT bundles formed by strong van der Waals interactions between the tubes stemming from a 1D structure similar to CNT bundles. Figure 1e is a high-resolution transmission electron microscope (HRTEM) image of SWBNNTs that were scarcely found in the sample synthesized at a nitrogen pressure of 14 bar, from which we were able to identify to an extent the Moiré pattern with a hexagonal structure^48,49^. Thus, we found that the SWBNNTs synthesized under optimized conditions had a highly crystalline hexagonal structure. Moreover, the high crystallinity of the BNNTs can also be confirmed through Raman analysis^50,51^. Figure 1f shows the high-resolution Raman spectrum of the raw BNNTs, where the black line is the raw data and the red line is the Lorentzian-line fit. We confirmed a sharp peak at 1368.8 cm^−1^ with a calculated FWHM of 28.8 cm^−1^, which is assigned to the E_2g_ mode in the BN structure related to in-plane motion^50,51^. The Raman results indicated that our BNNTs have a high crystallinity evidenced by the long lifetime of the E_2g_ phonon. Also, we think that the difference of FWHMs in the optical phonon of BNNTs compared to other BN materials might be a result of quantum confinement or random strain fluctuation^52–56^. Therefore, our analysis data are comparable to the FWHM (25 cm^−1^) and Raman peak position (1367 cm^−1^ to 1370 cm^−1^) of other highly crystalline BNNTs and h-BN^14,50,51^.

We performed an electron energy loss spectroscopy (EELS) analysis to clearly identify the structure and constituents of the BNNTs and found the boron peak near 191 eV and the nitrogen peak near 401 eV. The K-shell ionization edge was distinguishable from the boron peak and nitrogen position in the EELS spectrum^6,57^. This result was due to the π* and σ* peaks of the hybridized chemical bonds of boron and nitrogen^57^. In particular, the π* peak occurs when the 1s electron is transferred to the vacant π* antibonding orbital region^57^. Thus, the strong intensity of the π* peak in our results clearly indicated sp2 hybridization. The π* peak in our spectrum was also significantly sharped and higher in intensity compared with that of BNNTs synthesized by CVD^41^. Thus; we can conclude that our BNNTs have a tubular form with a hexagonal structure comprising sp^2^ hybridized boron and nitrogen atoms^6,14,34,37,57^.

Figures 1h,i and S1 show the atomic compositions of the raw BNNTs determined by X-ray photoelectron spectroscopy (XPS) analysis. The XPS peaks primarily occurred at 191.1 eV (boron) and 398.9 eV (nitrogen) with an atomic percentage ratio of boron to nitrogen of 1.1: 0.9, which was due to the existence of amorphous boron in the raw BNNTs. Generally, the B-O bond (191.8 eV) was observed due to the growth mechanism of other BNNT synthesis methods^37,42^. However, the high nitrogen pressure and the use of a bundle of boron fibers as a boron source in our HTP method appear to suppress the effect of residual oxygen and moisture which could remain on the powder form of source materials in the other methods.

As shown by the above analysis results, we were able to synthesize a large amount of BNNTs with high crystallinity at high pressure (14 bar, the optimized condition). However, fine control of structure such as BNNT walls and corresponding diameter is still required for specific applications. For this purpose, the growth kinetics of BNNTs were systemically studied. The growth pressure of the nitrogen feed gas was varied, while the growth temperature remained constant, which was confirmed by black body radiation measurements and calculations based on a heat loss model.

**Figure 2:**
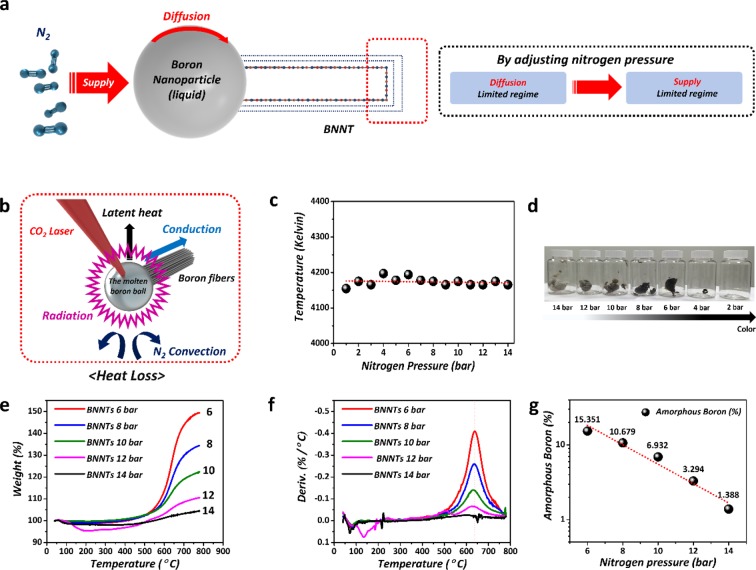
Growth mechanism model and analysis of BNNTs (**a**) BNNT growth mechanism model (diffusion-limited regime and supply-limited regime). (**b**) Heat loss model of the molten boron ball. (**c**) Measured absolute temperatures of molten boron ball under different N_2_ pressures from 1  to 14 bar. (**d**) Photo images of BNNTs synthesized under different pressures (2, 4, 6, 8, 10, 12, and 14 bar). (**e**) Increased weight (%) after the oxidation of various samples. (**f**) TGA plots (weight, %) under different N_2_ pressures. (**g**) TGA plots (derivative weight, %/°C) under different N_2_ pressures.

The growth mechanism can be understood based on the root growth model proposed by ONERA^58^. The tubular structure begins to grow from a pre-formed BN cap on the surface of molten boron nanoparticles, as shown in Figure 2a^58^. Nitrogen molecules, after adsorbing on the surface of the nanoparticles, undergo dissociation and subsequent surface diffusion to form BN species. Then, a BN structure can nucleate in the form of the BN cap, and continued growth takes place to form complete tubular structures.

For the fine structural control, understanding the growth kinetics of BNNTs is critical, and it can be considered in a way similar to that of CNTs where diffusion- and supply-limited kinetics regimes predominate^59^. At higher nitrogen pressures, the concentration of adsorbed nitrogen atoms on the surface is high, and thus diffusion of BN radicals toward the cap is a limited step. In this condition, extra caps can form and overlap on the preexisting BN cap, forming a multi-layered BN cap. From this point onward, the tubular structure will grow simultaneously from each layer of the BN cap, resulting in MWBNNTs. By decreasing the pressure, one can decrease the adsorption of nitrogen molecules onto the surface of the boron nanoparticles resulting in supply limited condition of BN radicals. The formation of extra BN caps will thus be suppressed, and nanotube growth from a single or double BN cap will subsequently be promoted due to the increasing diffusion rate of nitrogen species toward the cap. Because the supply of nitrogen molecules for BNNT growth decreases with decreasing pressure, the growth kinetics will make a transition from the diffusion-limited regime to the supply-limited regime.

The BNNT growth kinetics can be affected not only by the amount of nitrogen but also by the reaction temperature. Therefore, we evaluated the surface temperature of the molten boron ball using Wien’s displacement law and the observed black body radiation spectra under various pressures at a fixed laser power, as shown in Figure 2c. The results indicated that the absolute temperature was maintained at approximately 4175 kelvin regardless of the pressure conditions. We also conducted a theoretical approach to investigate the cause of the relative lack of temperature change using various equations to consider conduction, convection, latent heat loss, and radiation, as shown in Figure 2b and Table S1. Based on the equations in Table S1, we first confirmed that the estimated conductive energy loss was 0.198 W. Thus, conduction did not have a significant effect because the diameter of the boron fibers was very small. Second, the estimated convective energy loss was 8.482 W, a relatively low value indicating that convection was not the main path of cooling for the molten boron ball. Third, the estimated latent heat loss was 0.27 W. Latent heat loss also did not have a considerable effect because the feeding speed of the boron fibers was very slow in our experimental conditions.

On the other hand, the estimated radiative energy loss was 369.4 W. According to the Stefan-Boltzmann law, radiation becomes the main cooling path in the high-temperature region because the amount of radiant heat energy lost is proportional to the fourth power of the absolute temperature. Thus, radiation is the primary cooling path in our BNNT growth because the amount of radiant heat lost is independent of pressure. Consequently, our theoretical approach confirmed the experimental conclusion that the temperature of the boron ball was constant regardless of the nitrogen pressure in the chamber, and thus the pressure of nitrogen is the only factor that governs the kinetic regime.

Figure 2d is a set of photographs of BNNTs synthesized under various pressures from 14 bar to 2 bar. The raw BNNTs synthesized at 14 bar were almost light-gray because the amount of amorphous boron was significantly lower. However, the color of the synthesized raw BNNTs changed gradually from light-gray to black as the nitrogen pressure decreased. Even though we tried to synthesize the BNNTs at lower pressures than 6 bar several times, it was challenging to produce a large amount of BNNTs. We believe that it could be related to the growth kinetics of BNNTs. Therefore, we focused on the results prepared at the pressures from 6 bar to 14 bar in order to clearly compare and investigate the growth kinetics of BNNTs in HTP laser ablation.

Figure 2e–g show thermal gravimetric analysis (TGA) results of the BNNTs synthesized under various pressures. Generally, amorphous boron reacts with oxygen in the air to form boron oxide (B_2_O_3_) at temperatures over 500 °C. As a result, unlike carbon materials, the weight of the raw BNNTs including amorphous boron increased during TGA measurements. Figure 2e,f show the weight increase (%) and derivative values (%/°C) of the various samples after oxidation up to 800 °C. Based on the fact that weight increase occurred only in the pure amorphous boron sample and not in pure h-BN in a control experiment (refer to Figure S2a–d in the Supplementary Information), the amount of amorphous boron can be determined by the weight increase and chemical formula as presented in Figure 2g. Since the amount of amorphous boron in the raw sample is inversely proportional to the pressure of the nitrogen gas, the dissociation of nitrogen is presumed to be the rate-determining step in the two-step chemical reaction, $[{N}_{2(g)}\to {N}_{(g)}+{N}_{(g)}]\,$ and $[{B}_{(g)}+{N}_{(g)}\to B{N}_{(s)}]$, in our BNNT growth process.

Figure S3 shows FE-SEM images of the BNNTs synthesized at various pressures, which indicated that a large amount of BNNTs was synthesized at each pressure condition. As shown in Figure S3c, amorphous boron and a minimal amount of h-BN flakes were observed as impurities in our grown BNNTs through FE-SEM, which is similar to previous reports^34,37,45^. Figure S4 shows the HRTEM images of BNNTs synthesized under different pressure conditions, which corresponded to the change in BNNT color shown in Figure 2d. We also found that the morphologies and structures of the BNNTs synthesized at lower pressures were not distinctly different in terms of FE-SEM and TEM results despite the increase in the amount of amorphous boron.

**Figure 3:**
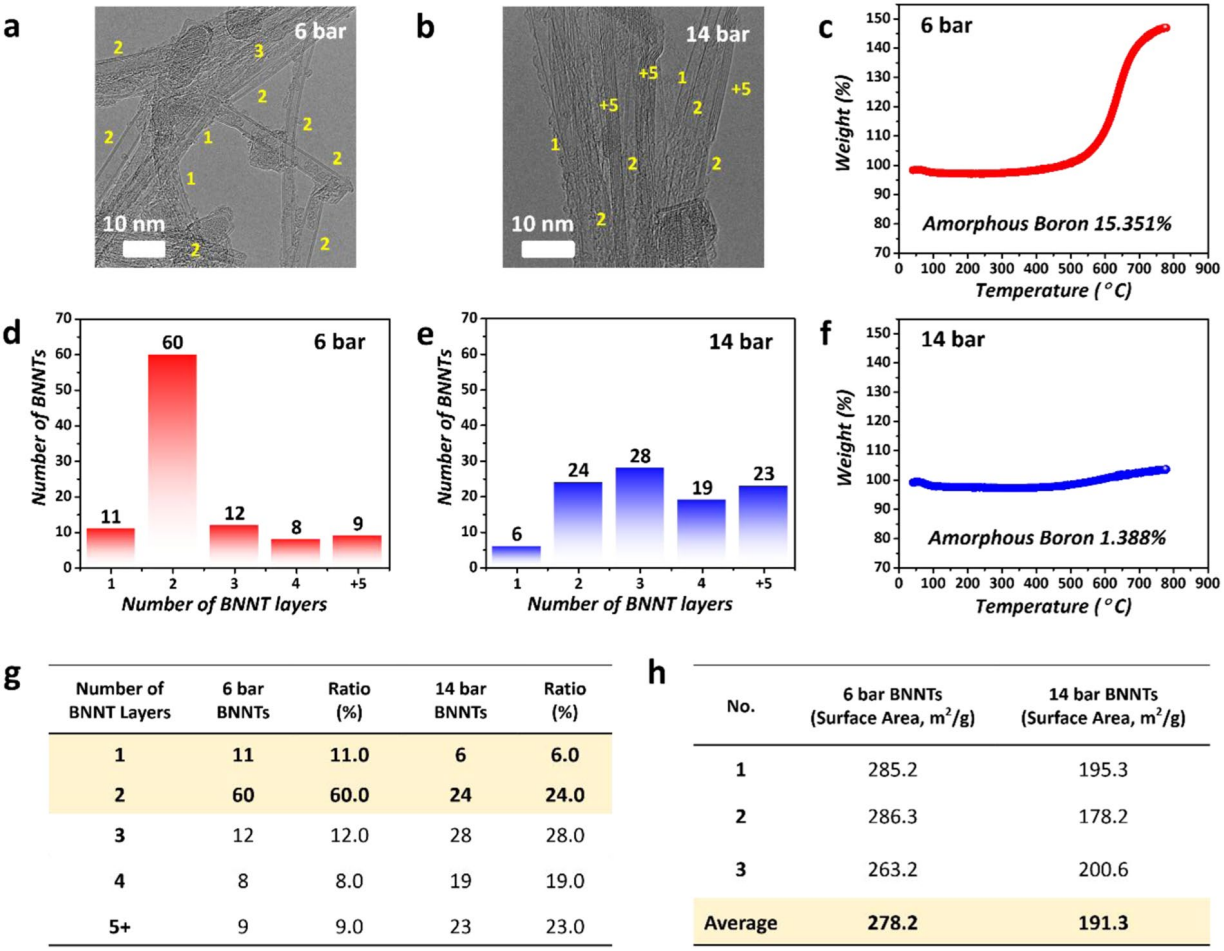
Comparison of BNNTs synthesized at 6 bar and 14 bar (**a,b**) TEM images of BNNTs synthesized at 6 bar and 14 bar. (**c**) TGA plot of BNNTs synthesized at 6 bar. (**d,e**) Numbers of BNNT layers synthesized at 6 bar and 14 bar. (**f**) TGA plot of BNNTs synthesized at 14 bar. (**g**) Table of numbers of BNNT layers synthesized at 6 bar and 14 bar. **h** BET analysis of BNNTs synthesized at 6 bar and 14 bar.

Figure 3a–h show a comparison of the properties of BNNTs synthesized under specific pressure conditions between 6 bar and 14 bar. As shown in Figure 3a–e, various SW, DW, and MWBNNTs were evenly synthesized at 14 bar, while SW and DWBNNTs were more frequently observed at 6 bar. This data analysis was performed based on 100 samples of BNNTs through TEM imaging (the same resolution) at 6 bar and 14 bar. As shown in Figure 3d,e,g, the total proportion of SW and DWBNNTs was evaluated as approximately 71% at a pressure of 6 bar, and most samples indicated the synthesis of DWBNNTs.

On the other hand, the total proportion of SW and DWBNNTs was approximately 30% at a pressure of 14 bar. Notably, it was confirmed that BNNTs with more than five layers comprised approximately 23% of the whole sample. It is interesting that DWBNNTs were synthesized to approximately 60% at a pressure of 6 bar; the main reason for this selective growth of DWBNNTs could be related to electrostatic interactions from AA′ stacking and the buckling effect, which stabilized the BN tubular structure^13,60–64^. DWBNNTs could be more easily stabilized than SWBNNTs during BNNT growth because the interaction of buckled lattices in DWBNNTs induces a stronger interlayer locking between the two walls^13,60–64^. The dominant production of DWBNNTs is consistent with the results of other previous reports that analyzed the wall number of BNNTs that were synthesized using similar methods^25,34,65,66^.

Figure 3c,f show the TGA results of BNNTs synthesized at 6 bar and 14 bar. In our experiments, when all the synthesis conditions except for the pressure were fixed, the amounts of amorphous boron synthesized at 6 bar and 14 bar were calculated as 15.351% and 1.388%, respectively. In particular, we believe that most of the boron source at 14 bar was used for the BNNT growth at the high reaction temperature during BNNTs synthesis. The mass increase due to oxidation was just 1.388% at 800 °C, as shown in TGA data at 14 bar.

Figure 3h shows Brunauer–Emmett–Teller (BET) measurements of the BNNTs synthesized at 6 bar and 14 bar. In particular, the average BET value of the BNNTs synthesized at 6 bar was 278.2 m^2^/g, and that of the BNNTs synthesized at 14 bar was 191.3 m^2^/g. Regarding the BET value of BNNTs, previous theoretical calculation and experimental papers reported that the BET value of SWCNTs was higher than those of other MWCNTs^67–69^. Thus, we can conclude that our high BET value at 6 bar arises from the existence of SW and DWBNNTs.

In order to prepare pure BNNTs for applications, we conducted a dry and wet purification process to remove amorphous boron (refer to Figure S5 in Supplementary Information). In the purification step, the raw BNNTs were oxidized in a furnace at 650 °C for 6 h, and the chemical reaction followed the formula $[2B+3{O}_{2}\to {B}_{2}{O}_{3}]$ to oxidize amorphous boron to B_2_O_3_^27,37,70–72^. The optimal oxidation temperature was chosen based on the TGA results in Figure 2e–g, and the exposure time was fixed to 6 h to remove amorphous boron. Since the produced B_2_O_3_ can be dissolved in methanol or water, it was physically separated and thus removed using bath sonication in methanol. As a result, pure BNNTs were collected by filtration. Figure S5a,b present the TEM images of amorphous boron before and after purification, which proved that the amorphous boron was entirely removed by our purification process.

**Figure 4:**
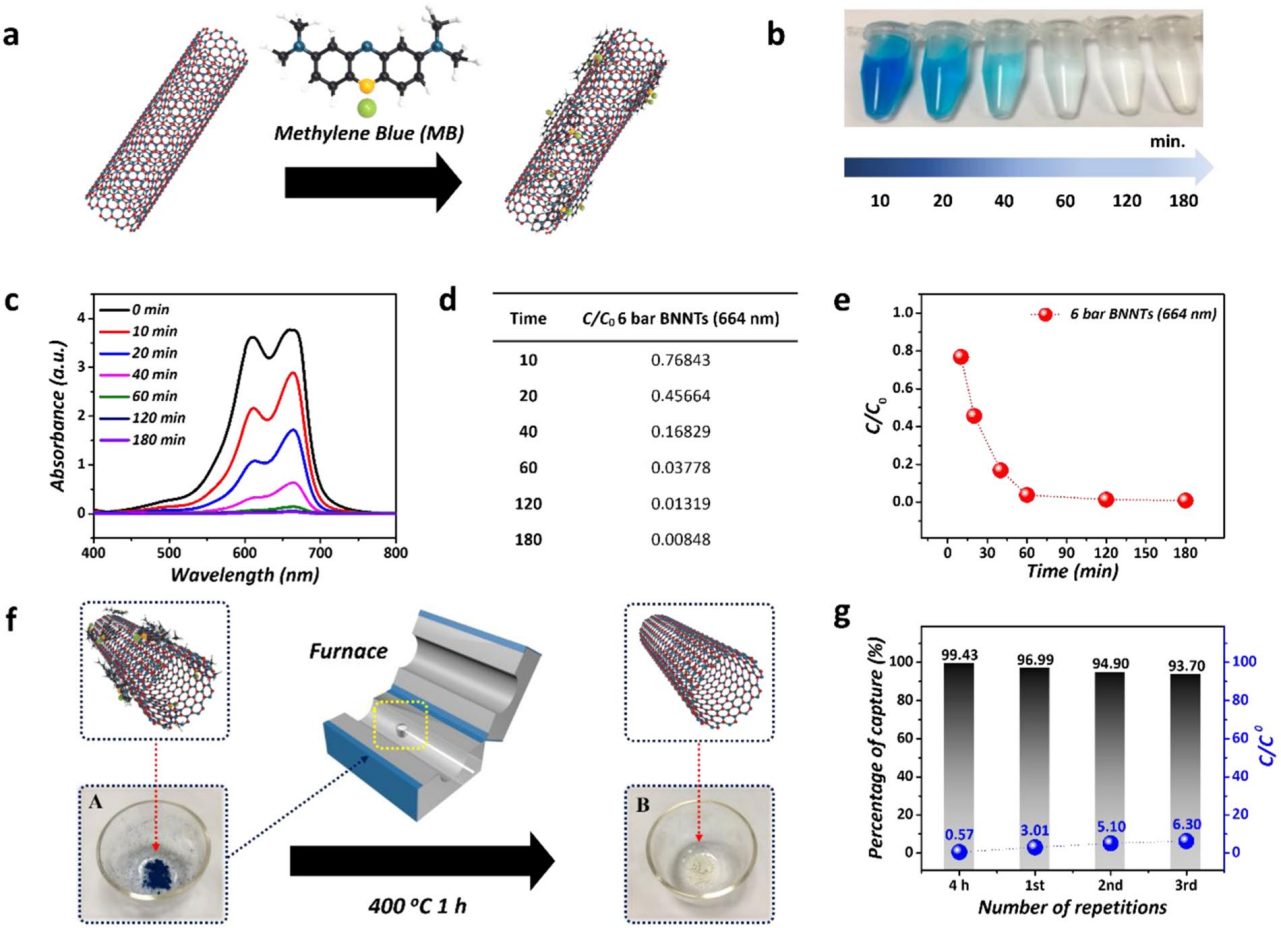
Application of BNNTs as adsorbents to selectively capture MB (**a**) Scheme of the reaction between BNNTs and MB. (**b**) Photo images of the color change resulting from the changing concentration of MB from 10 min. to 180 min. (**c**) UV-vis absorption spectra of the solution. (**d,e**) Different C/C_0_ values for 6 bar BNNTs depending on time. (**f**) Recycling process of BNNTs through the simple annealing method to remove MB. (**g**) Percentages of MB captured depending on repeated usages.

Recently, many researchers have been focused on the water purification because of the environmental issues^73–75^. Therefore, we applied to the application for water purification to take advantage of their high quality, surface area, and aspect ratio. Figure 4 presents the results of applying the BNNTs synthesized at 6 bar as adsorbents for water purification. The strong and fast adsorption might be caused by the structural similarities between the conjugated aromatic ring structure of the MB and the honeycomb BNNT structure. The π–π stacking interaction between MB and BNNTs can play a significant role in MB adsorption, as shown in Figure 4a. MB was selectively captured by the surface of BNNTs (adsorbent), as indicated by the color change of the solutions (Figure 4b) and the decrease in UV-vis absorption (Figure 4c) collected during water purification. The results were attributed to the effective MB adsorption characteristics of BNNTs. Considering the equation (refer to the Experimental section) and values of C/C_0_ in Figure 4c,d, we estimated 87% and 99% removal of MB from the solutions after 40 min and 180 min, respectively, despite the use of a low concentration of BNNTs/MB solution (10 mg/30 mL:30 ppm of MB). After 180 min, the C/C_0_ value was saturated about to zero, reflecting the fact that most of the MB molecules were removed. Figure 4f illustrates the reusability of the BNNT adsorbents for MB adsorption. The removal of MB from the BNNTs by simple combustion was carried out at 400 °C for 1 h in a furnace. After 3 repeated cycles of adsorption of MB and regeneration, approximately 93.7% of the MB was still removed by the recycled BNNT adsorbent. Our result of the reusability of the BNNTs and the high efficiency (approximately 94%) to repeatedly remove MB corresponds to the previously reported results (approximately more than 90%) of removing MB using other BN structures including bulk h-BN, thin h-BN, porous h-BN, 3D BN structures, and BN fibers^75–78^. From Figure 4g, we can conclude that the BNNTs synthesized at 6 bar could be promising materials for effectively removing MB from water in a recyclable manner.

## Conclusions

In summary, we studied the BNNT growth kinetics in HTP laser ablation synthesis. Considering the fact that nitrogen gas is supplied to and dissociates on boron nanoparticles and then diffuses to growth points, the growth kinetics can be classified into the diffusion-limited regime and the supply-limited regime depending on the pressure conditions. The growth temperature, which could affect chemical reactions including dissociation of nitrogen gas, was maintained as constant. This was confirmed by black body radiation experimental results and calculations based on a heat loss model including conduction, convection, radiation, and latent heat losses. In the supply-limited kinetics regime, we were able to selectively synthesize SW and DWBNNTs (71%) at a lower pressure (6 bar) compared with those (30%) at standard pressure (14 bar). Since SW and DWBNNTs have a large surface area, they could be used as a recyclable adsorbent to capture MB for water purification. Subsequent removal of MB was conducted by a simple annealing process at 400 °C for 1 h. Approximately 93.7% of MB was ultimately removed despite the three times repeated use of the BNNTs as an adsorbent. These selectively grown SW- and DWBNNTs could be promising materials for filters, high-performance polymer-composites, and bio-applications.

## Supplementary information

Supplementary information.

Supplementary Movie S1
