# Supplementary material for: Single- and double-walled boron nitride nanotubes: Controlled synthesis and application for water purification
Source: Sci Rep. 2020 May 4;10:7416. doi: 10.1038/s41598-020-64096-z (PMC7198605; doi:10.1038/s41598-020-64096-z)
Supplement: Supplementary file 1 — Supplementary information. [file 41598_2020_64096_MOESM1_ESM.docx]

***Supplementary Information***

Single- and double-walled boron nitride nanotubes: Controlled synthesis and application for water purification

*Hyunjin Cho^1,6,†^, Jun Hee Kim^1,2,†^, Jae Hun Hwang^1,3^, Cheol Sang Kim^2,3^, Se Gyu Jang^1^, Cheol Park^4^, Hun Su Lee^5,^*, and Myung Jong Kim^7,^**

^1^Functional Composite Materials Research Center, Korea Institute of Science and Technology, 92, Chudong-ro, Bongdong-eup, Wanju, Jeollabuk-do 55324, Republic of Korea

^2^Department of Bionanosystem Engineering, Chonbuk National University, 567, Baekje-daero, Deokjin-gu, Jeonju, Jeollabuk-do 54896, Republic of Korea

^3^Division of Mechanical Design Engineering, Chonbuk National University, 567, Baekje-daero, Deokjin-gu, Jeonju, Jeollabuk-do 54896, Republic of Korea

^4^Advanced Materials and Processing Branch, NASA Langley Research Center, Hampton, Virginia, 23681, USA.

^5^Mutifunctional Structural Composite Research Center, Korea Institute of Science and Technology, 92 Chudong-ro, Bongdong-eup, Wanju, Jeollabuk-do 55324, Republic of Korea

^6^Security and Disruptive Technologies Research Centre, National Research Council Canada, 1200 Montreal Road, Ottawa, Ontario K1A 0R6, Canada

^7^Department of Chemistry, Gachon University, 1342 Seongnam-daero, Sujeong-gu, Seongnam-si, Gyeonggi-do, 13120, Republic of Korea

KEYWORDS: BNNT; SWBNNT; DWBNNT; Growth mechanism; Water purification

†These authors contributed equally to this work.

*To whom correspondence should be addressed: [myung](mailto:myung)jongkim@gachon.ac.kr (Myung Jong Kim), [a123@kist.re.kr](mailto:a123@kist.re.kr) (Hun Su Lee)

**
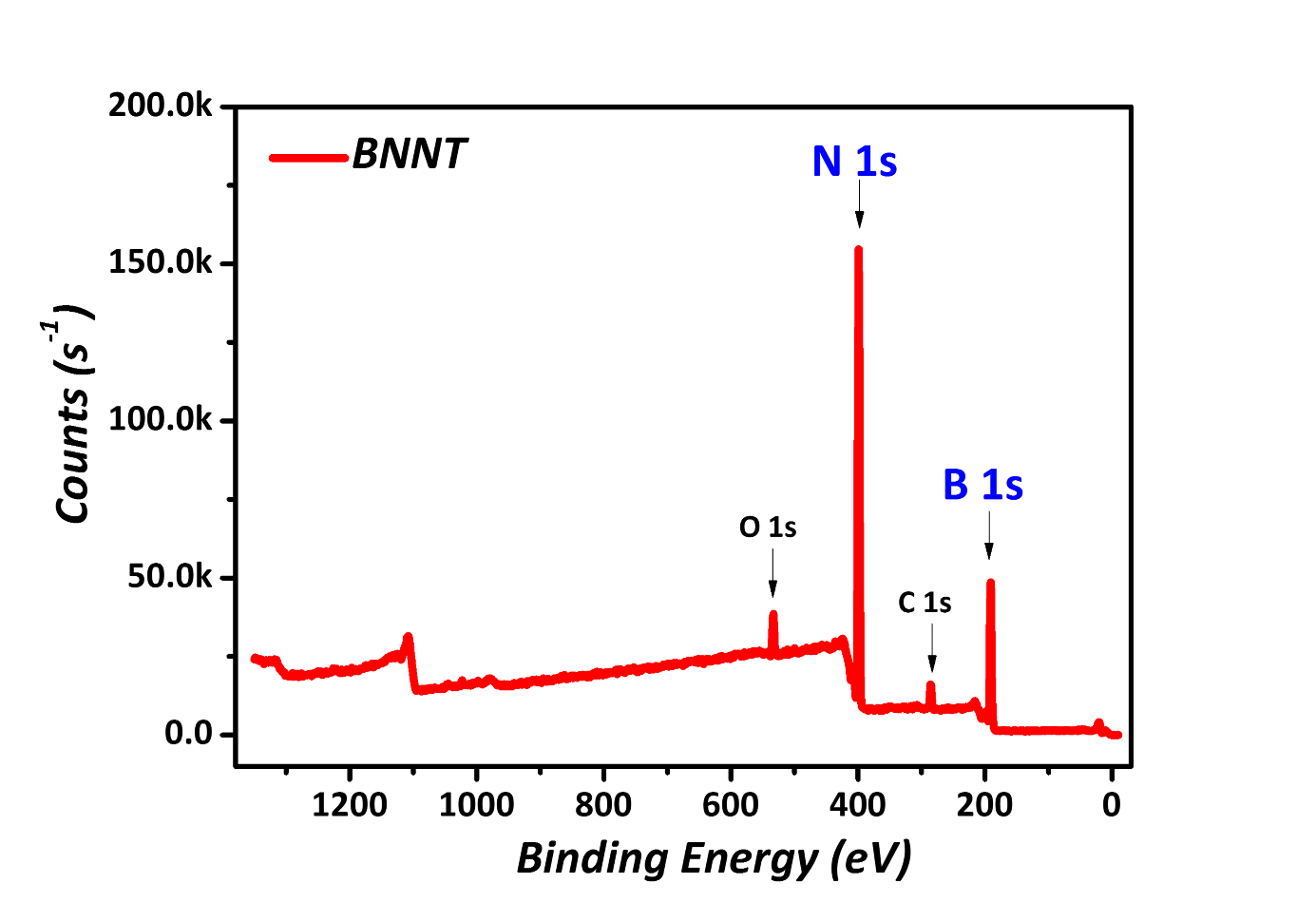
Fig. S1. XPS spectrum of BNNTs synthesized under 14 bar of N_2_ pressure.**

**
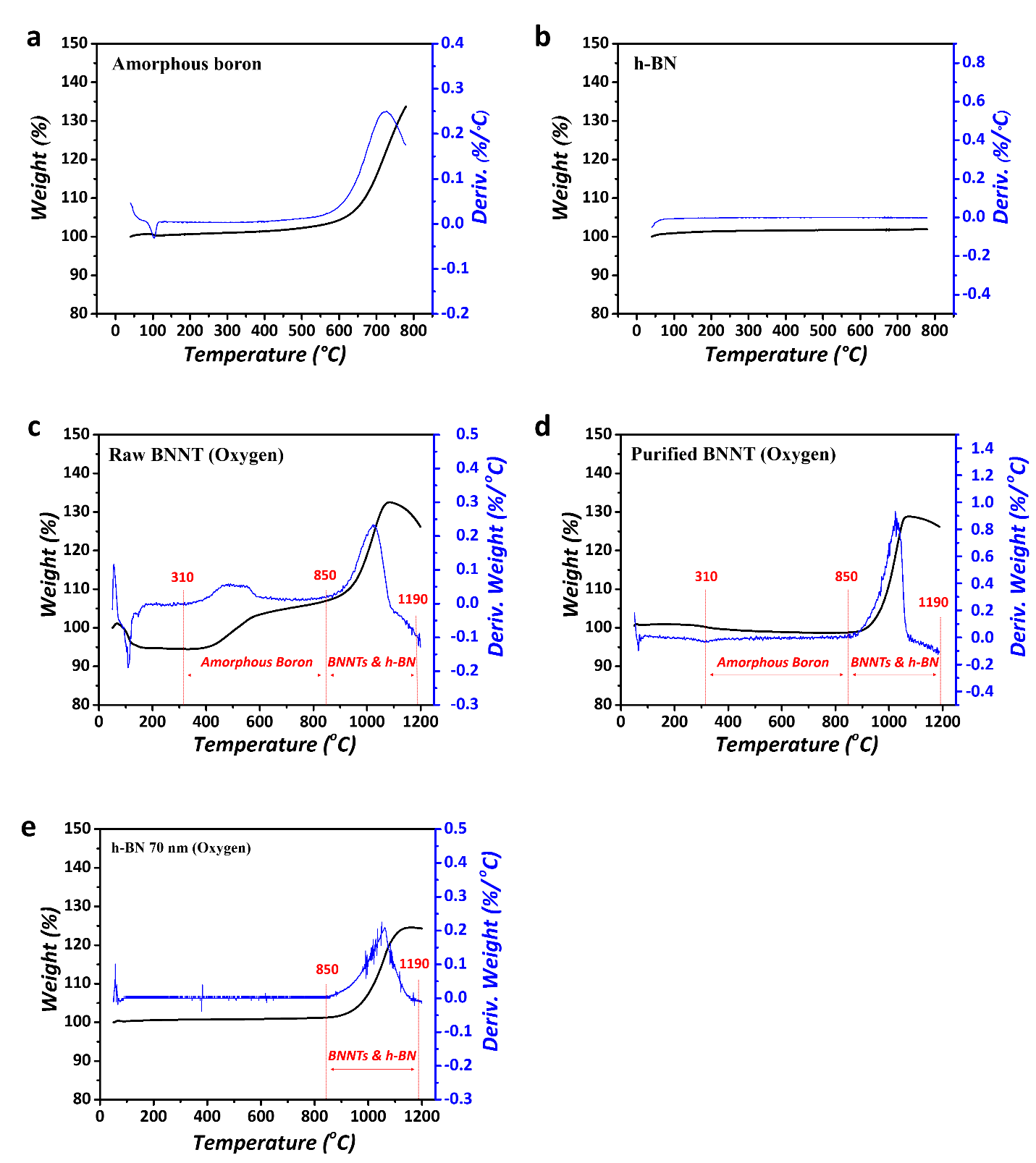
**

**Fig. S2. TGA plots (weight (%), derivative weight, (%/^o^C)) of h-BN and amorphous boron.**

**
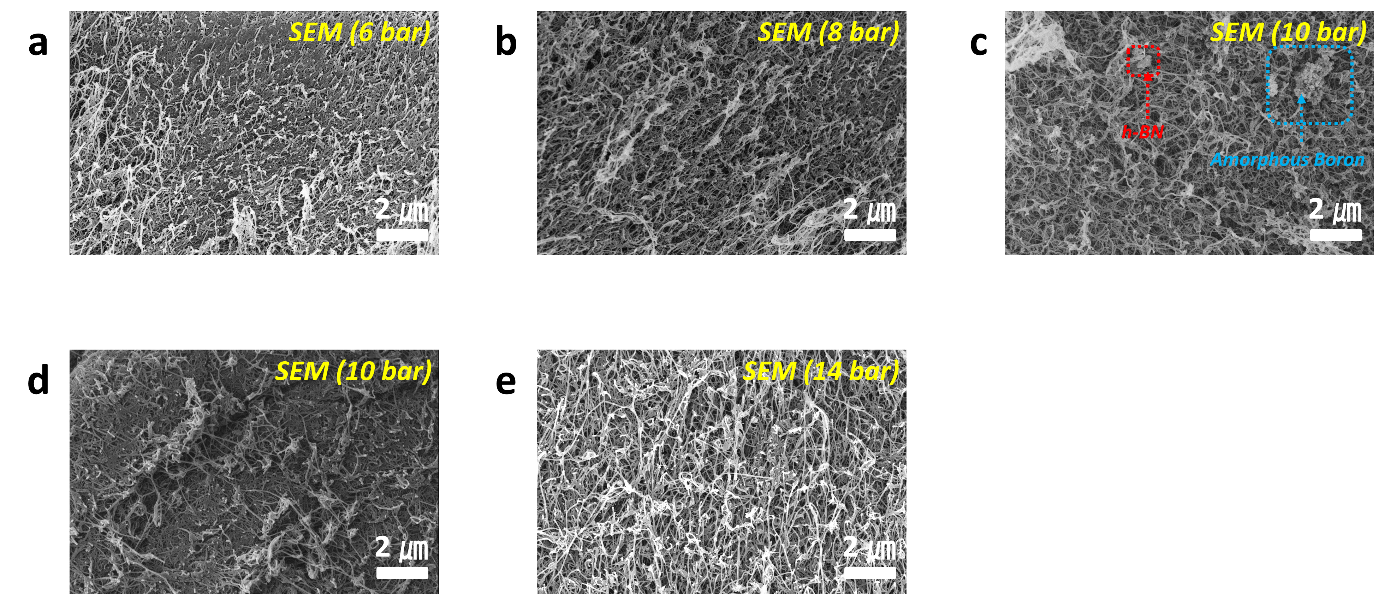
**

**Fig. S3. SEM images of BNNTs** **synthesized under N_2_ pressures from 14 bar to 6 bar.**

**
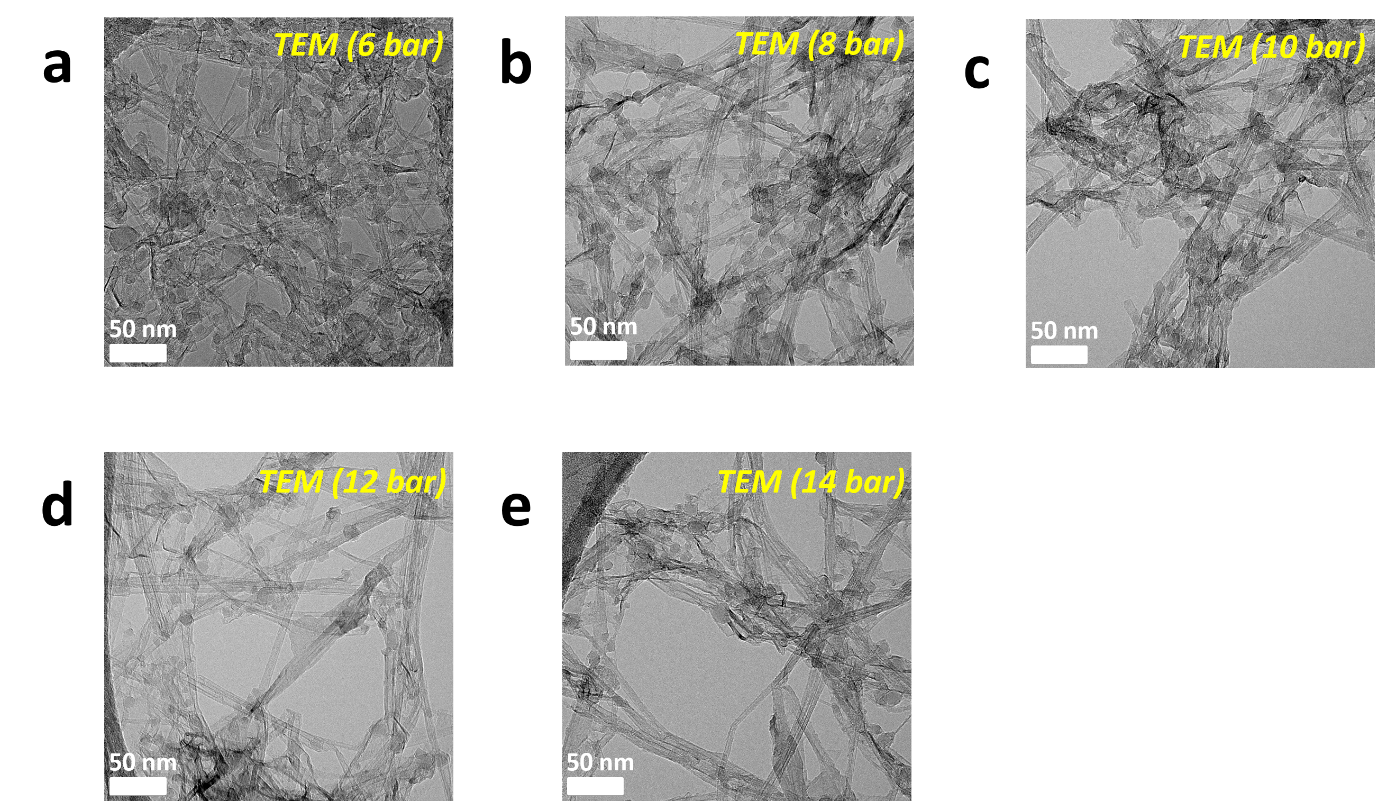
**

**Fig. S4. TEM images of BNNTs synthesized under N_2_ pressures from 14 bar to 6 bar.**


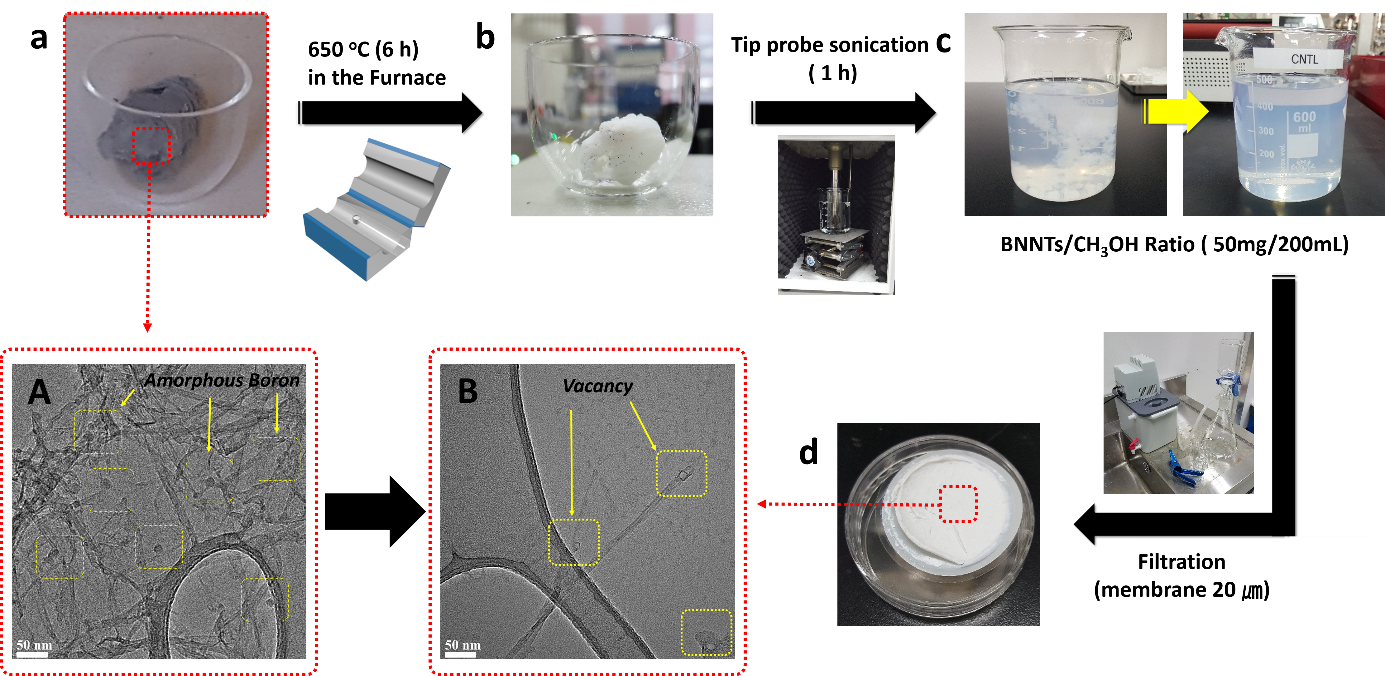


**Fig. S5. Purification process of BNNTs synthesized by HTP laser ablation.**

**
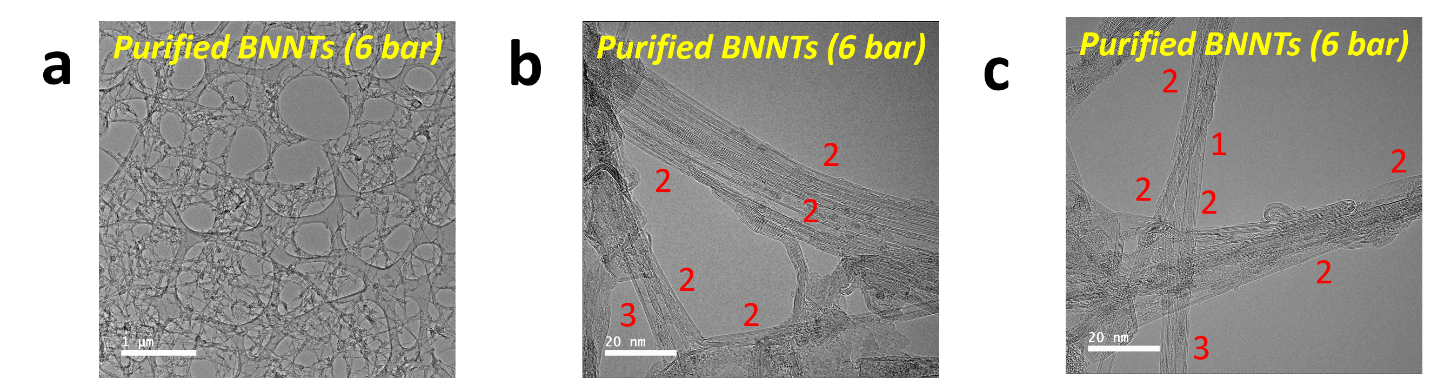
**

**Fig. S6. TEM images of Purified BNNTs (6 bar).**

**Table S1. Equations and measured values for evaluating the surface temperature of the molten boron ball.**

**
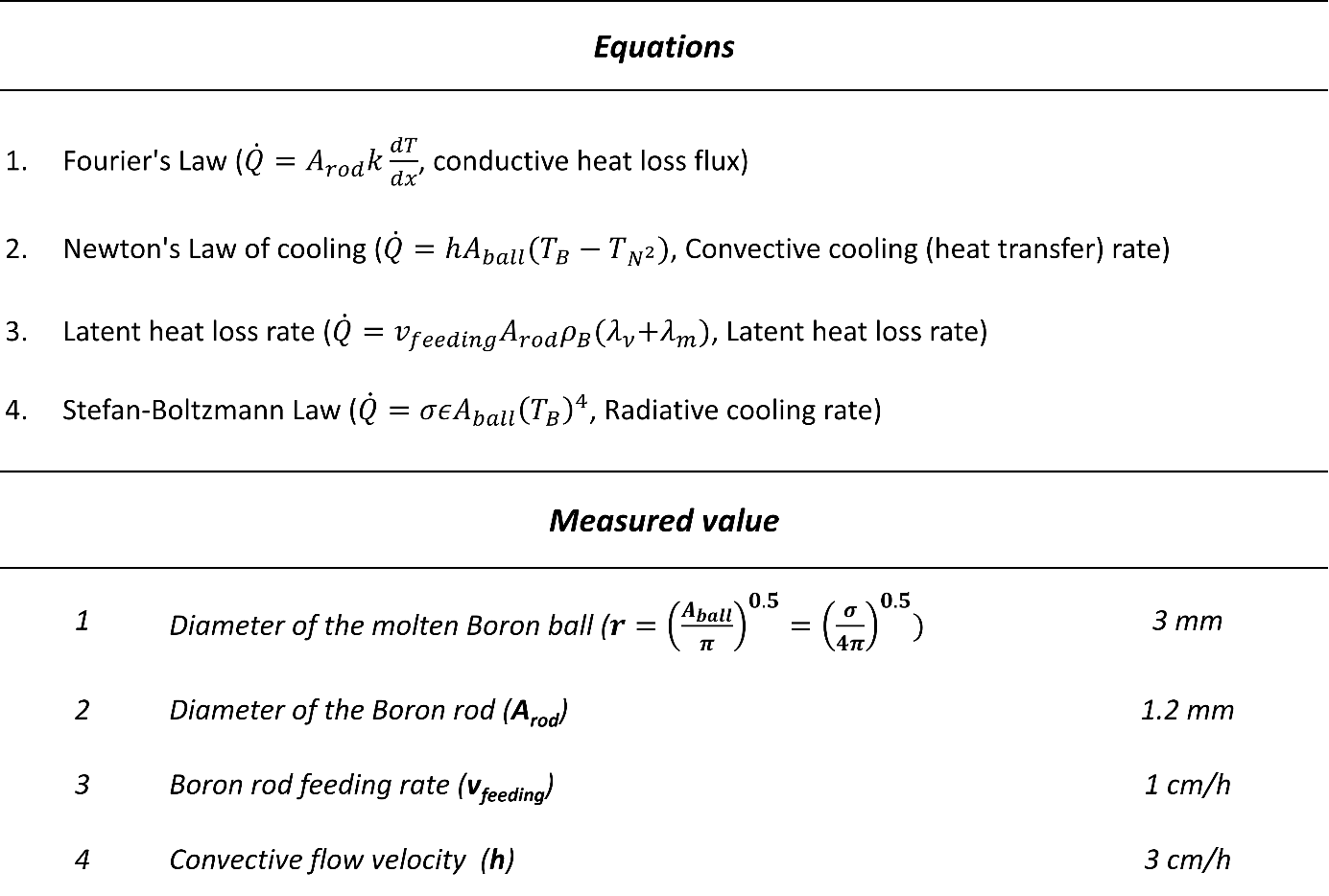
**
